# Clinically Meaningful Use of Mental Health Apps and its Effects on Depression: Mixed Methods Study

**DOI:** 10.2196/15644

**Published:** 2019-12-20

**Authors:** Renwen Zhang, Jennifer Nicholas, Ashley A Knapp, Andrea K Graham, Elizabeth Gray, Mary J Kwasny, Madhu Reddy, David C Mohr

**Affiliations:** 1 Department of Communication Studies Northwestern University Evanston, IL United States; 2 Center for Behavioral Intervention Technologies Feinberg School of Medicine Northwestern University Chicago, IL United States; 3 Department of Medical Social Sciences Feinberg School of Medicine Northwestern University Chicago, IL United States

**Keywords:** mHealth, mobile apps, mental health, engagement

## Abstract

**Background:**

User engagement is key to the effectiveness of digital mental health interventions. Considerable research has examined the clinical outcomes of overall engagement with mental health apps (eg, frequency and duration of app use). However, few studies have examined how specific app use behaviors can drive change in outcomes. Understanding the clinical outcomes of more nuanced app use could inform the design of mental health apps that are more clinically effective to users.

**Objective:**

This study aimed to classify user behaviors in a suite of mental health apps and examine how different types of app use are related to depression and anxiety outcomes. We also compare the clinical outcomes of specific types of app use with those of generic app use (ie, intensity and duration of app use) to understand what aspects of app use may drive symptom improvement.

**Methods:**

We conducted a secondary analysis of system use data from an 8-week randomized trial of a suite of 13 mental health apps. We categorized app use behaviors through a mixed methods analysis combining qualitative content analysis and principal component analysis. Regression analyses were used to assess the association between app use and levels of depression and anxiety at the end of treatment.

**Results:**

A total of 3 distinct clusters of app use behaviors were identified: learning, goal setting, and self-tracking. Each specific behavior had varied effects on outcomes. Participants who engaged in self-tracking experienced reduced depression symptoms, and those who engaged with learning and goal setting at a moderate level (ie, not too much or not too little) also had an improvement in depression. Notably, the combination of these 3 types of behaviors, what we termed “clinically meaningful use,” accounted for roughly the same amount of variance as explained by the overall intensity of app use (ie, total number of app use sessions). This suggests that our categorization of app use behaviors succeeded in capturing app use associated with better outcomes. However, anxiety outcomes were neither associated with specific behaviors nor generic app use.

**Conclusions:**

This study presents the first granular examination of user interactions with mental health apps and their effects on mental health outcomes. It has important implications for the design of mobile health interventions that aim to achieve greater user engagement and improved clinical efficacy.

## Introduction

Over the past decade, mobile phone apps have become portals for managing health. These digital tools help users monitor physical activity, plan healthy meals, and keep track of daily moods and other personal data. Given the accessibility and ubiquity of mobile phones, researchers and clinicians have increasingly leveraged mobile phone apps to deliver health interventions and enhance self-management of chronic conditions such as depression and anxiety [1-4]. Mental health apps can enhance skill building, deliver psychoeducation, and facilitate self-monitoring, thereby reducing symptoms of depression and anxiety [2,5,6]. These mobile technologies incorporate a wide array of system features and strategies that foster user engagement and promote behavior change, such as customization, reminders, self-monitoring, rewards, and peer support [3,7-10].

For mental health apps to be effective and successful, user engagement is critical. However, little consensus exists on how to define and measure engagement [11-13]. Engagement has been inconsistently viewed as a multidimensional construct, variably encompassing behavioral, emotional, and cognitive factors [14,15]. Consequently, engagement measures vary enormously, ranging from self-report questionnaires to system usage data or sensor data [16,17]. In this study, we define user engagement as a behavioral experience that involves people’s physical interaction with mobile phone apps [18]. System usage data are the most commonly used behavioral measures of engagement in mobile health (mHealth) interventions, as they can be acquired in the course of app use and require no additional effort from the user [14]. These data quantitatively capture how users physically interact with the app in terms of intensity (eg, number of app use sessions [19]), frequency (eg, number/percentage of days the app is used [20]), duration (eg, time spent on the app [21,22]), time between first and last app use [19,20]), and types (eg, passive, active, reflective, and didactic [23,24]). These measures of behavioral engagement have been found to be related to health outcomes such as psychological well-being [20,22].

However, behavioral engagement metrics have typically employed broad use metrics that measure the *quantity* of engagement. Few studies have examined more granular user interactions with specific components of mHealth interventions, which might provide insight into *how* a person is using an app in ways that are clinically meaningful [25]. For example, a user might simply open an app in response to a prompt without any deeper engagement, whereas others might engage in more meaningful activities such as inputting or reflecting on data and reading content. These different types of activities might reflect differing levels and types of engagement, resulting in various health outcomes. Identifying the types of user behaviors could provide insight into what aspects of mHealth interventions are engaging to users and what aspects may drive behavior change and symptom improvement [15,26]. This could also inform opportunities for designing more engaging and clinically effective mHealth interventions.

This study aimed to provide a categorization of the types of user behaviors in a suite of mental health apps for depression and anxiety. We then examine how the different types of app use are related to improvements in symptoms of depression and anxiety. To provide a holistic picture of app use, we also differentiate the more nuanced app use from generic app use (ie, intensity and duration of app use) and examine how these different use metrics influence outcomes. As such, this study presents the first granular classification of user interactions with mental health apps and their impact on outcomes.

## Methods

### Participants and Procedure

This study represents a secondary analysis of data from a randomized trial examining the efficacy of coaching and app recommendations to increase engagement with IntelliCare, a suite of mental health apps (Clinicaltrials.gov NCT02801877). Full study details have been described elsewhere [27]. In brief, participants were recruited between July 2016 and May 2017 via social and print media advertising, research registries, and commercial recruitment firms. People interested in participating completed an initial Web-based questionnaire deployed through a secure Web-based data capture system (Research Electronic Data Capture; [28]). The inclusion criteria were as follows: (1) aged 18 years or older, (2) reported elevated symptoms of depression (Patient Health Questionnaire-9 [PHQ-9] ≥10) or anxiety (generalized anxiety disorder-7 [GAD-7] ≥8), (3) resided in the United States, (4) could speak and read English, and (5) had an Android phone with data and text plans. Following baseline assessment, 301 eligible participants were randomized to 1 of the 4 treatments within a 2×2 factorial design for 8 weeks. Brief descriptions of the mobile intervention and each condition are provided below. The trial was approved by the Northwestern University Institutional Review Board before participant contact and monitored by an independent data safety monitoring board throughout the study period. All participants provided informed consent.

### Treatments

#### IntelliCare

The IntelliCare platform consists of 12 clinical apps, each targeting a specific behavioral or psychological treatment strategy (eg, cognitive restructuring, behavioral activation, social support, and relaxation) to improve symptoms of depression and anxiety. The specific apps have been described in more detail elsewhere [2,19]. In addition, a Hub app consolidates notifications from the downloaded clinical apps and is able to recommend other apps in the suite. IntelliCare apps prioritize interactive skills training over psychoeducation and are designed for frequent, short interactions.

#### Coaching

Participants assigned to the coach condition received 8 weeks of coaching aimed to support engagement. Coaching was based on a low-intensity coaching model [29] that relied primarily on brief SMS text messaging (2-3 messages per week) to promote engagement. Participants assigned to the self-guided condition had no sustained contact with coaches. Participants in both conditions received an orientation call at the beginning of the trial to ensure they had appropriately installed the Hub app and understood how to use the IntelliCare platform.

#### Recommendations

Participants randomized to the recommendation condition received recommendations for new apps weekly through the Hub app. The recommendation system leveraged app use data from approximately 80,000 users who had downloaded the IntelliCare apps to identify apps that the individual was more likely to use based on their app use profile. Participants not assigned to the recommendation condition did not receive recommendations and were encouraged to explore the apps by themselves.

### Measures

#### App Use Metrics

Usage logs for each app were recorded locally on the user’s mobile phone, which were then obtained and analyzed to extract app use metrics. In this study, we categorized 2 types of app use: clinically meaningful app use and generic app use.

**Figure 1:**
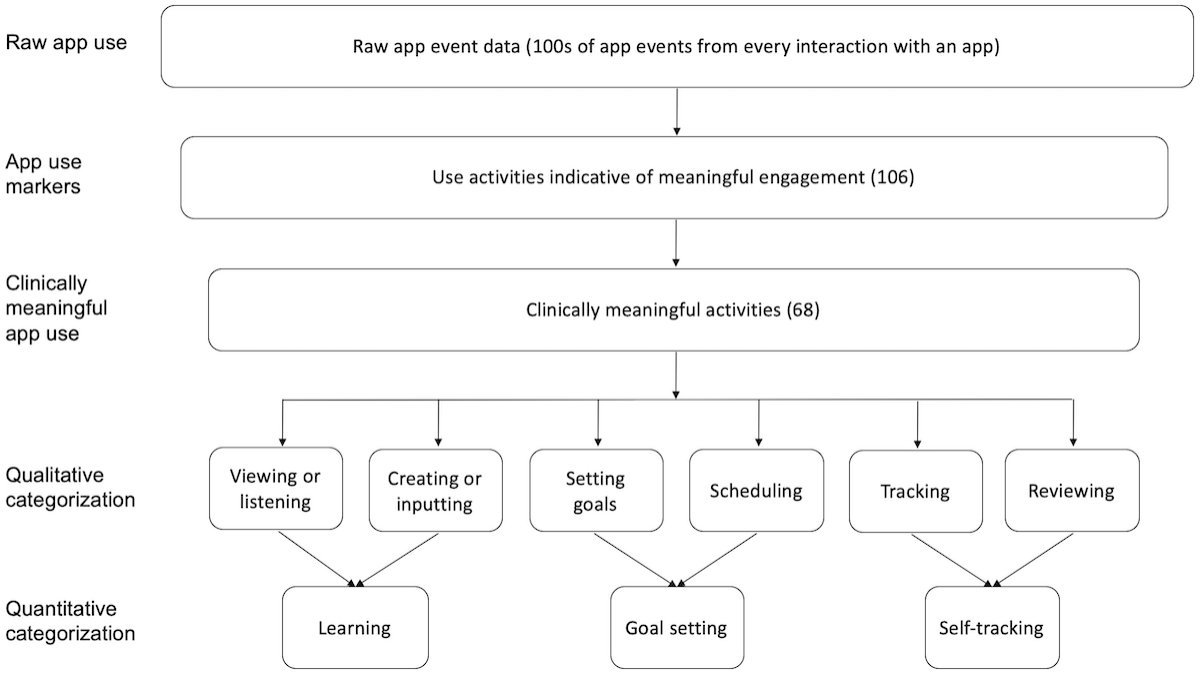
Procedure for categorizing app use activities across 13 IntelliCare apps.

*Clinically meaningful app use* metrics were developed in a 2-step process. First, a group of 5 psychologists who had been involved in the design of the apps created granular app use markers from raw event data. These app use markers were each defined by a small number of app event data considered to be indicative of meaningful engagement. Examples of such markers are app event data that defined completion of a skill-building exercise, reading psychoeducational text, or logging daily activities. Second, 2 authors (AZ and JN) developed a coding scheme to categorize these app use markers, drawing on existing literature and qualitative interviews with 32 IntelliCare users. Specifically, these 2 authors first collectively coded 18.8% (20/106) of the app use markers to develop the coding scheme and then used this coding scheme to separately categorize the remaining markers. Any disagreements in categorization were sent to the third author (AK), who served as a tiebreaker, and were resolved through rigorous discussion. During this process, we removed a number of app use markers that rarely occurred (eg, texting a friend from within the app) or were not considered clinically meaningful (eg, launching the app and viewing app use tips). Through this process, we identified 67 aggregated activities considered to be clinically meaningful, which we labeled “clinically meaningful use activities.” These activities were further grouped into 6 types (see Multimedia Appendix 1). The categorization procedure is presented in the bottom 4 steps of Figure 1. The 6 types of clinically meaningful activities identified were as follows:

1. Viewing/listening: reading/watching/listening to content from the app (eg, playing an exercise video, viewing a coping card, and listening to a relaxing audio)
2. Creating/inputting: creating and editing content for the purpose of learning and cultivating a skill (eg, identifying a coping activity and creating a positive or self-affirming statement)
3. Setting goals: selecting, editing, or adding self-identified or assigned goals (eg, adding or deleting a checklist item and selecting a weekly goal)
4. Scheduling: scheduling activities or changing reminders to fit one’s schedule (eg, scheduling an upcoming exercise and changing the reminder time)
5. Tracking: keeping track of one’s own performance or status through checking off, rating, or logging personal activities and moods, including facts and reasons (eg, checking a completed activity, rating a level of stress, and creating a sleep log)
6. Reviewing: reviewing one’s own content and progress (eg, reviewing past activities and lessons).

*Generic app use* was measured by 2 metrics: intensity of use and duration of use [22]. Intensity of use was defined as a user’s total number of app use sessions over the 8-week treatment period. An app use session was specified as a sequence of user-initiated actions or events separated by less than 5 min. Duration of use was defined as the total time an individual spent using the apps over the treatment period. It was calculated by summing the mean duration (in hours) of daily app sessions across all days in treatment.

#### Outcome Assessment

The primary outcomes of the study were depression and anxiety symptom severity, measured with the PHQ-9 [30] and GAD-7 [31] at baseline and end of treatment (week 8). Higher scores reflect higher levels of depression or anxiety.

### Data Analysis

Principal component analysis was performed on the 6 identified types of clinically meaningful activities, standardizing by type, to explore any underlying patterns of these activity types. Medians and IQRs of app use metrics were reported. Then, the relationship between app use metrics and treatment outcomes was analyzed using linear regression analyses, adjusting for baseline PHQ-9 or GAD-7 and randomization strata. We first plotted the bivariate relations between all use metrics and end-of-treatment outcomes, which revealed nonlinear patterns. In response, we categorized each app use metric into 4 quartiles. We considered the first quartile minimal intensity of use, the second quartile low intensity of use, the third quartile moderate intensity of use, and the fourth quartile high intensity of use. Regression models were fit to examine the relationship between the quartiles of app use metrics and outcomes, using the lowest quartile as the reference group. Regression coefficients (beta) with their 95% CIs and significance levels were reported for both unadjusted and adjusted models. In addition, the *R*^2^ values were reported for the unadjusted models to assess the magnitude of the effect. All analyses were performed using R version 3.5.1.

### Ethical Standards

The authors assert that all procedures contributing to this work comply with the ethical standards of the relevant national and institutional committees on human experimentation and with the Helsinki Declaration of 1975, as revised in 2008.

## Results

### Participants

A total of 301 eligible participants were enrolled in the randomized trial. The majority of participants were female (228/301, 75.7%), and the mean age was 37 (SD 11.84) years, ranging from 18 to 69 years. Most (237/301, 78.7%) of the participants identified themselves as white, 29 (9.6%) as African American, 10 (3.3%) as Asian, and 25 (8.3%) as “other.” The mean baseline level of depression (PHQ-9) was 13.21 (SD 4.63), and the mean baseline level of anxiety (GAD-7) was 11.98 (SD 4.02). A total of 10 participants discontinued treatment and were lost to follow-up. Further details of the sample and participant flow through the study are reported in the study by Mohr et al [27].

### Clinically Meaningful Use

**Figure 2:**
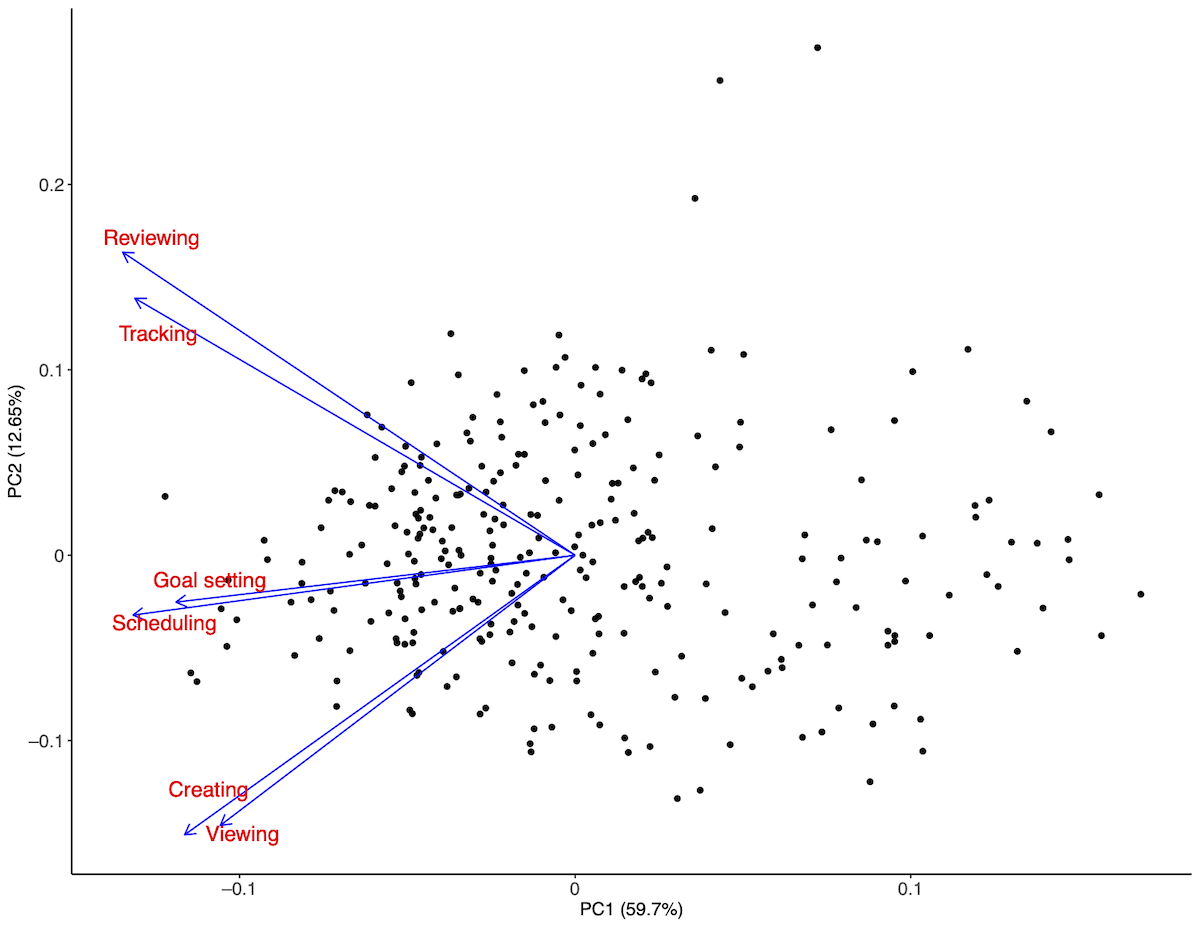
Principal component analysis of the types of clinically meaningful activities.

Correlation analysis showed that the 6 identified types of clinically meaningful activities were highly correlated; accordingly, we conducted a principal component analysis to further group these activity types. The analysis identified 3 clusters of meaningful activities that could best be described as: (1) “learning,” encompassing “viewing” and “creating;” (2) “goal setting,” including “setting goals” and “scheduling;” and (3) “self-tracking,” consisting of “reviewing” and “tracking.” The first 2 principal components explained 72.4% of the variability in the data (see Figure 2). Figure 1 presents a visual illustration of the categorization of clinically meaningful activities across the apps.

Self-tracking was performed most often, with a median frequency of 152 activities (IQR 61-300). Learning was performed less often, with a median frequency of 110 activities (IQR 52-191). Goal setting was used the least, with a median frequency of 59 activities (IQR 15-141). We also examined the frequency of overall clinically meaningful app use by combining all the 67 identified clinically meaningful use activities. The median frequency of clinically meaningful app use was 400 (IQR 200-608).

### Generic App Use

Over the 8-week treatment period, the median number of app use sessions was 184 (IQR 116-306), and the median duration of app use over the 8-week treatment period was 3.0 hours (IQR 1.7-5.0).

### App Use and Depression Outcome

#### Clinically Meaningful Use

We first examined how each of the 3 clusters of clinically meaningful activities predicted individuals’ level of depression at the end of treatment, compared with the lowest quartile (minimal use) of each cluster (Table 1). End of treatment PHQ-9, after controlling for treatment condition and baseline PHQ-9, was significantly related to moderate intensity of learning (beta=−2.17; *P*=.002); moderate intensity of goal setting (beta=−2.08; *P*=.007); and low, moderate, and high intensity of self-tracking (beta=−2.46; *P*=.002; beta=−1.94; *P*=.01; and beta=−1.92; *P*=.009, respectively), compared with minimal intensity use. Moreover, learning and control variables accounted for 29.2% variance of the model (adjusted *R*^2^=0.292), goal setting and control variables accounted for 26.5% variance of the model (adjusted *R*^2^=0.265), and self-tracking and control variables accounted for 27.4% variance of the model (adjusted *R*^2^=0.274).

**Table 1:** Regression models of 3 clusters of clinically meaningful activities predicting depression outcome.

Covariate		Model 1^a^		Model 2^b^		Model 3^c^	
		Estimate (SE)	*P* value	Estimate (SE)	*P* value	Estimate (SE)	*P* value
Intercept		1.86 (0.93)	.047	1.57 (0.93)	.09	2.16 (0.91)	.02
Coached		−0.78 (0.52)	.13	−0.42 (0.56)	.46	−0.12 (0.56)	.83
Full Hub		−0.21 (0.50)	.67	−0.28 (0.51)	.59	−0.05 (0.51)	.93
PHQ9_baseline		0.54 (0.05)	<.001	0.56 (0.06)	<.001	0.55 (0.06)	<.001
**Learning_minimal intensity^d^**							
	Learning_low intensity	0.64 (0.73)	.39	—^e^	—	—	—
	Learning_moderate intensity	−2.17 (0.71)	.002	—	—	—	—
	Learning_high intensity	−1.22 (0.73)	.09	—	—	—	—
**Goal setting_minimal intensity^d^**							
	Goal setting_low intensity	—	—	−0.62 (0.76)	.41	—	—
	Goal setting_moderate intensity	—	—	−2.08 (0.76)	.007	—	—
	Goal setting_high intensity	—	—	−0.76 (0.76)	.32	—	—
**Self-tracking_minimal intensity^d^**							
	Self-tracking_low intensity	—	—	—	—	−2.46 (0.78)	.002
	Self-tracking_moderate intensity	—	—	—	—	−1.94 (0.76)	.01
	Self-tracking_high intensity	—	—	—	—	−1.92 (0.73)	.009

^a^*R*²=0.307; Adjusted *R*²=0.292.

^b^*R*²=0.281; Adjusted *R*²=0.265.

^c^*R*²=0.289; Adjusted *R*²=0.274.

^d^Values of reference group.

^e^Not applicable.

In addition to examining the 3 identified clusters of clinically meaningful activities, we also explored how outcomes were related to overall clinically meaningful app use. As shown in Table 2 (model 1), after controlling for treatment condition and baseline level of depression, PHQ-9 at the end of treatment was significantly and negatively associated with low intensity of meaningful use (beta=−2.00; *P*=.007), moderate intensity of meaningful use (beta=−2.07; *P*=.006), and high intensity of meaningful use (beta=−2.05; *P*=.006), compared with minimal intensity of clinically meaningful app use. Clinically meaningful app use and the control variables accounted for 27.3% variance of the model (adjusted *R*^2^=0.273).

#### Generic App Use

PHQ-9 at the end of treatment was significantly and negatively associated with low intensity of generic app use (beta=−1.44; *P*=.047), moderate intensity of generic app use (beta=−2.38; *P*=.001), and high intensity of generic app use (beta=−2.45; *P*=.001), compared with minimal intensity of generic app use (Table 2, model 2). In general, as the number of generic app use sessions increased, symptoms of depression at the end of treatment decreased. The intensity metrics and control variables accounted for 27.9% variance of the model (adjusted *R*^2^=0.279). Only moderate duration of use was significantly associated with PHQ-9 at week 8 (beta=−1.52; *P*=.045) when compared with minimal duration of use (Table 2, model 3). The duration metrics and control variables accounted for 25.8% variance of the model (adjusted *R*^2^=0.258).

**Table 2:** Regression models of total meaningful app use, generic app use, and duration of app use predicting depression outcome.

Covariate		Model 1^a^		Model 2^b^		Model 3^b^	
		Estimate (SE)	*P* value	Estimate (SE)	*P* value	Estimate (SE)	*P* value
Intercept		2.25 (0.92)	.02	2.24 (0.92)	.02	1.71 (0.93)	.07
Coached		−0.34 (0.53)	.52	−0.58 (0.51)	.26	−0.57 (0.54)	.23
Full Hub		−0.11 (0.51)	.82	0.26 (0.54)	.63	0.02 (0.53)	.98
PHQ9_baseline		0.55 (0.06)	<.001	0.55 (0.05)	<.001	0.54 (0.06)	<.001
**Meaningful use_minimal intensity**							
	Meaningful use_low intensity	−2.00 (0.74)	.007	—^e^	—	—	—
	Meaningful use_moderate intensity	−2.07 (0.74)	.006	—	—	—	—
	Meaningful use_high intensity	−2.05 (0.74)	.006	—	—	—	—
**Generic app use_minimal intensity^d^**							
	Generic app use_low intensity	—	—	−1.44 (0.72)	.047	—	—
	Generic app use _moderate intensity	—	—	−2.38 (0.73)	.001	—	—
	Generic app use_ high intensity	—	—	−2.45 (0.76)	.001	—	—
**Generic app use_minimal duration^d^**							
	Generic app use_low duration	—	—	—	—	−0.32 (0.75)	.68
	Generic app use_moderate duration	—	—	—	—	−1.52 (0.76)	.045
	Generic app use_high duration	—	—	—	—	−1.24 (0.78)	.12

^a^*R*²=0.288; Adjusted *R*²=0.273.

^b^*R*²=0.295; Adjusted *R*²=0.279.

^c^*R*²=0.274; Adjusted *R*²=0.258.

^d^Values of reference group.

^e^Not applicable.

### App Use and Anxiety Outcome

Anxiety (GAD-7) at the end of the treatment was neither significantly associated with the 3 clusters of clinically meaningful activities (all *P*s>.11) nor the overall clinically meaningful app use (all *P*s>.13). Therefore, no further analyses were conducted regarding the association between anxiety and additional generic app use metrics.

## Discussion

### Principal Findings

This study provided a categorization of user behaviors in a suite of mental health apps and investigated how different types of app use were related to improvements in depression and anxiety symptoms following an 8-week intervention. The results showed that different types of clinically meaningful activities (ie, learning, goal setting, and self-tracking) had varied effects on outcomes. Self-tracking at varied levels of intensity was related to improvement in depression symptoms, whereas only moderate intensity of learning and goal setting predicted improvement in depression symptoms. Thus, this study provides insight into how different types of app use might be conducive to improved intervention outcomes.

Drawing on a mixed methods approach, we identified 6 types of clinically meaningful activities across multiple apps, which were further grouped into 3 clusters—learning, goal setting, and self-tracking. This categorization was achieved through a combination of qualitative content analysis and quantitative statistical analysis. The results show that users engaged in self-tracking most frequently, followed by learning and goal setting. These 3 types of use activities have been well documented in mHealth and human-computer interaction (HCI) research as approaches to drive engagement and promote behavior change [8,32]. However, little is known about how these activities are related to health outcomes, as previous studies have primarily focused on the clinical outcomes of generic app use [21,33]. By shifting attention from generic app use to a more granular examination of meaningful app use, this study provides a more nuanced understanding of user engagement with mental health apps.

Notably, overall clinically meaningful app use (combination of all 67 identified clinically meaningful use activities) accounted for roughly the same amount of variance in depression severity as explained by the intensity of overall app use (ie., total number of app use sessions). Therefore, our identification of clinically meaningful app use was successful at capturing the activities associated with better mental health outcomes. This suggests that we accurately identified the clinically meaningful intervention components within this suite of apps. As such, we believe that the association between app use and outcome can be largely explained by these clinically meaningful use activities, which clustered into 3 types of activities, reinforcing the importance of self-tracking, goal setting, and psychoeducation elements within mHealth interventions for depression.

More specifically, these 3 clusters of clinically meaningful activities were associated with reductions in depression symptoms at the end of treatment. In particular, self-tracking was found to be beneficial at all levels of intensity compared with minimal intensity of use. This is in accordance with HCI research suggesting that self-tracking, or personal informatics, can lead to behavior change [34], chronic disease management [35], and self-knowledge and self-reflexivity [36,37]. Our study extends this line of research by demonstrating the clinical benefits of self-tracking in the context of mental health. It is important to note that self-tracking in our study consisted of both data collection (ie, tracking) and data reflection (ie, review), as delineated in the stage-based model of personal informatics systems [34]. This suggests that mHealth could better support users by facilitating self-tracking through the incorporation of design features that promote data collection and data reflection.

It is important to note that greater amounts of engagement did not necessarily lead to greater reductions in depression. Although self-tracking was generally beneficial, only a moderate level of engagement with learning and goal setting was associated with reduced depressive symptoms. Neither high nor low intensity of app use could predict better outcomes compared with minimal intensity of use. This result suggests that mHealth interventions might follow the Goldilocks principle—“Not too much. Not too little. Just right” [38]. Just as in many digital technologies, mental health apps do not promise that “the more engagement, the better outcomes;” rather, we argue that the benefits of app use may only be seen when the doses of various classes of intervention features “just right.” Too frequent engagement with goal setting and learning may lead to fatigue, whereas too scarce engagement may lead to ineffectiveness. Thus, mental health technologies should be designed to promote use at the right amount, possibly by sending users reminders based on their app use data. An alternate explanation is that people who engage in learning and goal setting more frequently may be less responsive to treatment. That is, perhaps higher engagement in these activities is associated with a more treatment resistant course of depression, or is exhibited by individuals with more complex comorbidities, thereby indicating that the intervention components do not fit the needs of certain individuals. Thus, high engagement in these activities could be an indicator of risk of lower responsiveness to treatment and could be used to guide the implementation of alternative treatment strategies for individuals who are likely to benefit from additional support.

The overall intensity of generic app use also predicted reductions in depression symptoms. Generally, it appears that people who engaged in higher intensity of app use had lower levels of depression at the end of treatment. However, the duration of app use minimally contributed to better outcomes. This finding corresponds to prior work suggesting that people tend to use mobile apps in very short bursts of time, given their habit of using smartphones in spare moments [39,40]. Although duration of engagement plays a critical role in Web-based interventions [21], the current ways in which people interact with smartphones suggest that mHealth apps should be designed to be quick to use, have simple interactions, and support a single or limited set of related tasks [2,40]. IntelliCare aligns with these endeavors to facilitate frequent but short interactions.

However, our investigation of meaningful app use was not associated with reduced anxiety symptoms. This is consistent with the findings in the main trial, where significant reductions in anxiety symptom were not related to number of app sessions or time between first and last app use but were only associated with the number of app downloads [27]. The discrepancy in effects of app use on depression and anxiety is a novel finding and suggests that different types of use may be more effective for some psychological states and not others. We speculate that motivation may be an important factor. Users in low motivational states may require self-tracking, goal setting, and learning features in specific doses such that they receive enough to be beneficial but not so much that it overwhelms them. On the other hand, anxiety may be less sensitive to dose responses because it is a more activating condition. To understand such nuance, we need more research specifically designed to examine the relationship between clinically meaningful activities and symptoms across various disorders or symptomatology.

Overall, this study has important implications for the design of mHealth for depression, which includes the following:

- Self-tracking, goal setting, and learning are 3 components that have clinical benefits, which should be incorporated into mental health apps.
- Mental health apps could be designed according to the Goldilocks principle, incorporating the “just right” amount of intervention components and promoting use at the right amount, possibly through sending user reminders or alerts based on app use data.
- People tend to use apps in very short bursts of time, so mental health apps should be quick to use, have simple interactions, and support a single or limited set of related tasks.

However, because of the exploratory nature of the research, design considerations derived from this study focus only on app content and engagement. Within the wider context, research indicates that app design and quality assessment must also consider users’ lived experience, app usability and stability, and data privacy and security [41]. For example, the critical importance of privacy and security in relation to mental health apps was highlighted in 2 recent studies, which suggest that currently available mental health apps often misuse user data [42] and that users’ willingness to share personal sensing data varies depending on the type of data collected and with whom they are shared [43]. Indeed, the importance of these factors is evident within the ever-growing array of app quality measures and guidelines, including those from the American Psychiatric Association [41] and the US Food and Drug Administration [44].

### Limitations

Despite its contributions and implications, this study has some limitations. First, the user activities identified in this study were not exhaustive; some activities were eliminated because of their low frequency. As a secondary analysis, this study is exploratory by nature, and future studies should continue exploring more specific types and patterns of user behaviors in using mHealth technologies and their relationships with outcomes of mental health conditions. Second, although this study demonstrated the associations of both generic and specific app use with clinical outcomes over the treatment period, it is difficult to make causal claims about the effects. The relationship between app use and symptom change is likely dynamic. For example, app use may contribute to lower subsequent symptoms, and symptom changes may in turn increase app use [45]. Experimental studies are warranted to examine how different intervention components uniquely contribute to outcomes. Third, this study only examined outcomes within the 8-week treatment period. Future research could build on this preliminary model to explore the long-term effects of different app use behaviors.

### Conclusions

Engagement with digital health interventions is a long-standing problem; however, little is known about how users interact with mental health apps in clinically meaningful ways. This study employed a novel, mixed methods methodology to derive greater understanding of users’ engagement with apps that cannot be seen through generic use data. Using a combination of qualitative and quantitative methods, we uncovered 3 clusters of clinically meaningful activities—learning, goal setting, and self-tracking—with each type associated with reductions in depression symptoms. However, different activities and intensities of use produced varied effects. Although only moderate intensity of learning and goal setting led to reductions in symptoms of depression, self-tracking at all levels of intensity predicted improvement in depression. Understanding the relationship between different types of user activities and clinical outcomes could inform the design of mental health apps that are more clinically effective for users.

## Appendix

Multimedia Appendix 1 Codebook of the categorization of app use behaviors.

## Abbreviations

GAD-7: generalized anxiety disorder-7
HCI: human-computer interaction
mHealth: mobile health
PHQ-9: Patient Health Questionnaire-9
